# Urinary calprotectin as a diagnostic tool for detecting significant bacteriuria

**DOI:** 10.1038/s41598-024-62605-y

**Published:** 2024-05-28

**Authors:** Sabina Waldecker-Gall, Christoph B. Waldecker, Nina Babel, Xenofon Baraliakos, Felix Seibert, Timm H. Westhoff

**Affiliations:** 1 Rheumazentrum Ruhrgebiet, Ruhr-University Bochum, Herne, Germany; 2 Department of Nephrology, St. Marien-Hospital Mülheim an der Ruhr, Mülheim, Germany; 3 Center for Translational Medicine, University Hospital Marien Hospital Herne, Ruhr University Bochum, Herne, Germany; 4 Medical Department 1, University Hospital Marien Hospital Herne, Ruhr-University Bochum, Hölkeskampring 40, 44625 Herne, Germany

**Keywords:** Calprotectin, Bacteriuria, Biomarker, Urinary tract infection, Immunology, Microbiology, Biomarkers, Health care, Nephrology, Urology

## Abstract

Pyuria in dipstick examination serves as the most widespread screening tool for urinary tract infections (UTI). The absence of pyuria, however, does not exclude UTI. We investigated the diagnostic value of urinary calprotectin, a mediator protein of the innate immune system, which is released by leukocytes, for the detection of UTI and compared it with dipstick pyuria. Since even low numbers of leukocytes in the urine significantly increase urinary calprotectin concentrations, calprotectin might be a more sensitive marker than pyuria detected by dipstick. All 162 patients were prospectively included and underwent a urine dipstick, urine culture, quantification of proteinuria and determination of calprotectin in the urine. Urinary calprotectin was determined using an enzyme-linked immunosorbent assay (ELISA). UTI was defined as urine cultures with detection of one or a maximum of two uropathogenic bacteria with ≥ 10^5^ colony-forming units per millilitre (CFU/ml). Exclusion criteria were acute kidney injury, chronic renal insufficiency and tumors of the urinary tract. 71 (43.8%) patients had a UTI. Of the 91 patients without UTI, 23 had a contamination and 19 had evidence of ≥ 10^5^ CFU/ml considered to be asymptomatic bacteriuria. The median calprotectin concentration in patients with UTI and pyuria was significantly higher than in patients with UTI and without pyuria (5510.4 vs. 544.7 ng/ml). In ROC analyses, calprotectin revealed an area under the curve (AUC) of 0.70 for the detection of significant bacteriuria. Pyuria in dipstick examinations provided an AUC of 0.71. There was no significant difference between these AUCs in the DeLong test (p = 0.9). In patients with evidence of significant bacteriuria but without pyuria, a significantly higher calprotectin concentration was measured in the urine than in patients with neither pyuria nor UTI (544.7 ng/ml vs 95.6 ng/ml, p = 0.029). Urinary calprotectin is non-inferior to dipstick pyuria in the detection of UTI.

## Introduction

Urinary tract infections (UTIs) are among the most frequently treated bacterial infections in both ambulatory and inpatient care^1^. UTI is proven when there is microbiological evidence of bacterial colonisation of the urinary tract (renal pelvic caliceal system, ureter, bladder, urethra) with corresponding clinical symptoms such as dysuria, fever, imperative need to urinate, pollakiuria, flank pain. The diagnosis of a UTI is the most common reason for prescribing antibiotics in outpatients, with most infections being treated empirically. Most UTIs occur in otherwise healthy, sexually active, young adult women, where anatomical and lifestyle factors predispose them to UTI. However, while simple UTIs can usually be successfully treated with empirically prescribed antibiotics on an outpatient setting, patients with additional risk factors often require targeted antibiotic treatment. Asymptomatic bacteriuria (ABU) must be distinguished from UTI^2^. In ABU, there are no clinical symptoms, but bacterial colonisation of the urinary tract can be detected by means of a urine culture. This requires the detection of ≥ 10^5^ colony-forming units (CFU)/ml of urine in two consecutive urine cultures in women and in one in men if the patients are asymptomatic^3,4^. ABU is common and occurs in examinations of non-pregnant, healthy women and patients with anatomical and functional changes in the urinary tract. Only exceptions such as pregnant women or patients undergoing invasive procedures on the urinary tract should be treated for ABU^5^. The gold standard for the diagnosis of UTI is detection of bacteria in urine culture^6^. Significant bacteriuria in a UTI is defined by counts of > 10^5^ colony forming units per millilitre (CFU/ml) and > 10^4^ CFU/ml, in the midstream urine (MSU) of women and men, respectively^6^.

Urine culture, however, is time-consuming, expensive and does not allow a diagnosis to be made before the following day after urine collection^7^. Therefore, the most widespread screening tool is dipstick examination searching for pyuria. This method is inexpensive, rapid and ubiquitously available. In the majority of otherwise healthy patients with typical symptoms of UTI detection of pyuria in dipstick examination makes urine culture dispensable. Sensitivity of this technique ranges between 72 and 97% and the specificity between 41 and 86% in the literature^8^.

Calprotectin is a calcium-binding complex of two proteins of the so-called S100 group (S100A8/S100A9). It is released predominantly from neutrophils and, to a less extent, from monocytes^9^. In neutrophil cytoplasm it adds up to 60% of the cytosolic proteins^10^. Calprotectin is an activator of the innate immune system. If a neutrophil granulocyte is stimulated by an invading microorganism, it releases calprotectin as a damage-associated molecular pattern proteins (DAMP). Calprotectin activates toll-like receptor 4 (TLR4) and thereby amplifies inflammation^11^. With regard to its origin and physiological role calprotectin constitutes a promising biomarker candidate for UTI. In analogy, faecal calprotectin is an established biomarker in gastroenterology to detect inflammatory bowel conditions. Since calprotectin is released by neutrophils in high concentrations, it appears possible that it is a more sensitive marker of bacteriuria than a dipstick examination. The present study compares the diagnostic accuracy of urinary calprotectin concentrations and dipstick pyuria in the detection of UTI.

## Methods

### Study population and design

We conducted a prospective cohort study on inpatients recruited from University Hospital Marien Hospital Herne, tertiary care internal medicine and nephrology center at Ruhr-University Bochum, Germany. Patients were included in the study if a UTI was clinically suspected or if they submitted a urine sample for other reasons, such as renal end organ damage in the case of arterial hypertension or diabetes mellitus or in the case of an unclear increase in infection parameters. In accordance with the primary endpoint, a particular focus was placed on recruiting patients with a leucocyte-negative urine status and a positive urine culture including asymptomatic patients. In order to identify these patients, the laboratory values of all inpatients at our internal medicine clinic were screened. The respective medical history, the clinic and the presence of elevated laboratory parameters typical of infections such as C-reactive protein (CRP) or leucocytes as well as the dipstick/urine culture helped to identify potential patients. Exclusion criteria were acute kidney injury (AKI), chronic renal insufficiency and tumors of the urinary tract.

When analysing the data, both an analysis of the entire study population and a subgroup analysis of patients with and without a UTI were performed. UTI was defined as urine culture with detection of one or a maximum of two uropathogenic bacteria with ≥ 10^5^ CFU/ml^6^. Urine cultures with evidence of more than two uropathogens were regarded as a contamination. The control group therefore consisted of patients with no evidence of bacteria in their urine and patients who had an asymptomatic bacteriuria.

The reporting of this study conforms to the Strengthening the Reporting of Observational Studies in Epidemiology (STROBE) statement^12^, which is presented in the supplementary material. Written informed consent was obtained from all participants before entry into the study. The study was performed by the Declaration of Helsinki and approved by the Ruhr-University Bochum ethics committee (ethics committee reference number: 5019-14).

### Laboratory measurements

Patients provided 3 urine samples 10 ml each from MSU for examination; catheter urine was obtained from patients who had an urinary catheter. The sample for the measurement of calprotectin concentrations was stored in a freezer (− 80 °C) until measurement, the second urine sample was examined directly after acquisition from the patient in the hospital laboratory (dipstick, quantification of proteinuria using a protein- and albumin-creatinine quotient in the spontaneous urine) and the third sample was used for microbiological examination within the urine culture following the usual processing technique. For the detection of pyuria, urine dipstick analysis Combur-Test 10® from Roche^13^ was used without microscopic examination and for the detection and identification of uropathogenic bacteria, solely urine culture was performed without the use of a Polymerase chain reaction-based molecular assay^14^. In order to exclude bias of the results of the two groups due to antibiotic treatment, the inhibitor test was used^2^. The determination of the protein- or albumin-creatinine quotient (PCR, ACR) is a less time-consuming and simpler test compared to the 24-h urine collection. Spontaneous urine is utilised for this purpose, which allows the test to be performed at the time of the patient's examination. Albumin, protein and creatinine are quantified in the spontaneous urine and an indication in mg/l is obtained. Protein and albumin are then set in relation to the creatinine excretion for the respective quotient and an indication in mg/g creatinine is obtained. Many studies have shown that the quantification of proteinuria using PCR is almost equivalent to the determination in 24-h urine collections. The determination of PCR for the investigation of proteinuria and for monitoring chronic kidney disease has been included in the KDIGO guidelines^15^. A PCR of > 200 mg/g creatinine is the cut-off value for detecting proteinuria^15^. PCR/ACR serves to exclude renal insufficiency in this study. The determination of the urinary calprotectin concentration was performed using enzyme-linked immunosorbent assay (ELISA) kit (PhiCal® Calprotectin, catalog number K 6928, Immundiagnostik AG, Bensheim, Germany) according to the manufacturer’s protocol^16,17^. Creatinine, CRP and leucoyte count were determined in all patients on hospitalisation by validated standard clinical blood tests of serum samples respectively Ethylenediamine tetraacetic acid (EDTA).

### Statistical analysis

The available data were checked for normal distribution using a D'Agostino-Pearson test^18^. This was not the case for the analysed parameters in the present population. The results were therefore presented in median and interquartile range (IQR). A Mann–Whitney *U* test was used to compare the data of patients with a UTI with those of patients without a UTI^19^. The null hypothesis stated that there was no difference between the two groups. A difference was considered significant at p < 0.05. ROC analysis was used to determine the diagnostic value of calprotectin with regard to the presence of a UTI in comparison with pyuria and to define a cut-off value for the detection of a UTI^20^. The Youden test was used to determine the cut-off value^21^. The area under the curve (AUC) of calprotectin and pyuria, which was calculated using ROC analysis, was compared using the DeLong test^22^. Furthermore, the sensitivity and specificity as well as the positive and negative predictive value for the detection of a UTI were calculated using calprotectin, the calprotectin-creatinine quotient and pyuria. The statistical programmes SPSS Version 26 (SPSS Inc, Chicago, Illinois, USA) and Excel® 2011 (Microsoft Corporation, Redmond, USA) were used for the analysis.

## Results

### Patient demographics, serum laboratory values and clinical characteristics of the study population

The demographic and clinical characteristics of the study population are presented in Table 1. A total of 162 patients were included. No kidney transplant patients were recruited. Of these, 66.67% (n = 108) were women and 33.33% (n = 54) were men. The median age was 67 years (IQR of 53.3–81.0 years), whereby the patients in the group with UTI (n = 71) were significantly older than those without UTI (n = 91) (p < 0.001). The median creatinine was 0.8 mg/dl (IQR of 0.7–0.9 mg/dl). In the subgroup analysis, there was no significant difference in relation to this value (p = 0.632). The median CRP value was 1.95 mg/dl (IQR of 0.5–6.3 mg/dl). In the group of patients with UTI, the CRP value was significantly higher (p = 0.018). Overall, 28.4% (n = 46) patients had one or more typical complaints (dysuria 4.94%, pollakisuria 3.09%, fever 24.69%). Contrary to our expectations, there was no significant difference in clinical symptoms between patients with UTI and those without UTI. A subgroup analysis of patients with and without UTI showed that 3 patients with UTI and 5 patients without UTI had dysuria (p = 0.701), 1 patient with UTI and 4 patients without UTI had pollakisuria (p = 0.272), while 22 patients with UTI and 18 patients without UTI had fever (p = 0.110). The proportion of those who had given MSU was significantly higher in the group without UTIs; the opposite was found for urine samples collected via a catheter (p = 0.02).

**Table 1:** Characteristics of the study population.

Variables	Total patients 162, (100%)	Patients with UTIs 71, (43.82%)	Patients without UTIs 91, (56.18%)	*p*-value*
Age in years (median, IQR)	67.0 53.3–81.0	76.0 62.0–84.5	60.0 47.5–72.0	< 0.001
Females n, (%)	108 (66.67%)	54 (76.06%)	54 (59.34%)	0.002
Males n, (%)	54 (33.33%)	17 (23.94%)	37 (40.66%)	
Creatinin mg/dl (median, IQR)	0.8 0.7–0.9	0.8 0.7–1.0	0.8 0.7–0.9	0.632
CRP mg/dl (median, IQR)	1.95 0.5–6.3	2.70 0.7–7.6	1.20 0.31–5.9	0.018
Midstream urine sample, (%)	125 (77.16%)	48 (67.61%)	77 (85.62%)	0.009
Catheter urine sample n, (%)	37 (22.84%)	23 (32.39%)	14 (15.38%)	
Dysuria n, (%)	8 (4.94%)	3 (4.23%)	5 (5.49%)	0.701
Pollakisuria n, (%)	5 (3.09%)	1 (1.41%)	4 (4.4%)	0.272
Fever n, (%)	40 (24.69%)	22 30.99%	18 (19.78%)	0.110

*UTIs* urinary tract infections, *CRP* C reactive protein, *IQR* interquartile range.

*p-value between patients with and without UTIs from Mann–Whitney-*U*-Test.

### Outcomes of the chemical urine analysis

Table 2 provides an overview of the results of the urine diagnostics (dipstick, calprotectin concentrations and PCR/ACR quotient) in the populations with and without UTI. The analysis of the urine dipstick comparing the subpopulations with and without UTI confirmed the results described in the previously cited literature. Proteinuria, pyuria and nitrite were significantly more frequent in the population with UTI (p < 0.05). When analysing pyuria further, it became apparent that there was only a significant difference in pronounced pyuria (500/µl) between the two groups (p < 0.05). In the lower range (25/µl, 75/µl and 100/µl), there was no significant difference between the population with and without UTI (p > 0.05). With regard to hematuria, there was no significant difference between the two groups (p > 0.05). The calprotectin concentration was found to be significantly higher overall and in relation to the creatinine concentration in the urine in patients with UTIs compared to those without UTIs (p < 0.001).

**Table 2:** Results of the biochemical urine analysis.

Variables	Total patients 162, (100%)	Patients with UTIs 71, (43.82%)	Patients without UTIs 91, (56.18%)	*p*-value*
Calprotectin in ng/ml (median, IQR)	1758.5 242.4–6260.4	3811.9 1102.5–8241.2	787.8 86.6–3253.0	< 0.001
Calprotectin/Creatinin in ng/mg (median, IQR)	2859.9 384.3–10,414.5	4637.0 1634.8–22,820.6	887.9 109.9–5044.8	< 0.001
Nitrites n, (%)	32 (19.75%)	22 (30.99%)	10 (10.99%)	0.002
Pyuria n, (%)	103 (63.58%)	59 (83.10%)	44 (48.35%)	< 0.001
Pyuria 25/µl n, (%)	25 (15.43%)	13 (18.31%)	12 (13.19%)	0.372
Pyuria 75/µl n, (%)	9 (5.56%)	3 (4.23%)	6 (6.59%)	0.515
Pyuria 100/µl n, (%)	9 (5.56%)	4 (5.63%)	5 (5.49%)	0.969
Pyuria 500/µl n, (%)	60 (37.04%)	39 (54.93%)	21 (23.08%)	< 0.001
Hematuria	87 (53.70%)	43 (60.56%)	44 (48.35%)	0.123
Proteinuria in Urinanalysis n, (%)	64 (39.51%)	39 (54.93%)	25 (27.47%)	< 0.001
PCR in mg/g Creatinin (median, IQR)	139 74.7–299.5	222 99.3–399.0	89.90 62.9–203.5	< 0.001
ACR in mg/g Creatinin (median, IQR)	29.5 5.0–97.8	64.0 7.7–143.0	11.5 3.8–54.7	< 0.001

*UTIs* urinary tract infections, *PCR* Protein/Creatinin ratio, *ACR* Albumin/Creatinin ration, *IQR* interquartile range.

*p-value between patients with and without UTIs from Mann–Whitney-*U*-Test.

### Outcomes of the microbiological urine analysis

The microbiological examination yielded the results shown in Table 3. The higher bacterial counts (10^5^ and 10^6^/ml) were significantly more frequent in the population with a UTI (p < 0.001 and p = 0.011) and, in contrast, lower bacterial counts (10^3^ and 10^4^/ml) were significantly more frequent in the group without a UTI (p = 0.002). A bias in the results of the two groups due to antibiotic treatment could be ruled out by the inhibitor test. In total, only 23 (22.33%) of the patients received an antibiotic treatment before undergoing the urine test. The frequency was the same in both groups (p = 0.385). The microbiological examination of the urine samples revealed, in addition to uropathogenic bacteria, several non-pathogenic bacteria which were interpreted as contamination. Furthermore, the frequency of contamination was specified, as these were categorised as patients without UTI despite the detection of bacteria. Accordingly, the number of contamination in the group without UTI is significantly higher (p < 0.001). In this study, E. coli was identified as the most common uropathogen for the development of a UTI with 41.96%. It was followed in descending frequency by coagulase-negative Staphylococci, Klebsiella pneumoniae and Enterococcus faecalis. The uropathogens were always detected significantly more frequently in the population with UTI (p < 0.005). The results of the differentiation of uropathogens in the microbiological urine analysis are summarised in Table 4.

**Table 3:** Results of the microbiological urine analysis.

Variables	Total patients 162, (100%)	Patient with UTIs 71, (43.82%)	Patients without UTIs 91, (56.18%)	*p*-value*
Bacterial detection n, (%)	103 (63.58%)	71 (100%)	32 (35.16%)	< 0.001
No bacterial detection	59 (36.42%)	0	59 (64.84%)	
CFU/ml 10^6^ n, (%)	8 (4.94%)	7 (9.86%)	1 (1.1%)	0.011
CFU/ml 10^5^ n, (%)	82 (50.62%)	64 (90.14%)	18 (19.78%)	< 0.001
CFU/ml 10^4^ n, (%)	11 (6.79%)	0	11 (12.09%)	0.002
CFU/ml 10^3^ n, (%)	11 (6.79%)	0	11 (12.09%)	0.002
Positive antimicrobial substances n, (%)	23 (22.33%)	12 (16.90%)	11 (12.09%)	0.385
Contamination (> 2 Bacteria) n, (%)	32 (19.75%)	0	32 (35.16%)	< 0.001

*UTIs* urinary tract infections, *CFU* colony forming units, *IQR* interquartile range.

*p-value between patients with and without UTIs from Mann–Whitney-*U*-Tests.

**Table 4:** Bacterial differentiation in microbiological urine analysis.

Variables	Total patients with bacterial detection 103, (100%)	Patients with UTI with bacterial detection 71 (43.82%)	Patient without UTIs with bacterial detection 32 (56.18%)	*p*-value*
*E. coli* n, (%)	47 (41.96%)	46 (64.79%)	1 (1.1%)	< 0.001
Coagulase- negative *Staphylococci* n, (%)	13 (11.61%)	10 (14.08%)	3 (3.30%)	0.012
*Klebsiella pneumoniae* n, (%)	6 (5.36%)	5 (7.04%)	1 (1.1%)	0.048
*Enterococcus faecalis* n, (%)	6 (5.36%)	6 (8.45%)	0	0.005

*UTIs* urinary tract infections;

*p-value between patients with and without UTIs from Mann–Whitney-*U*-Test.

### Comparison of calprotectin concentration in different subgroups

The present analysis confirmed that the more leukocytes in the urine, the increased concentration of calprotectin can be measured. In case of a high count of leukocytes in the urine (500/µl), a significantly higher concentration of calprotectin could be detected in the urine compared to a low leukocyte count (25/µl) (p < 0.001). When the calprotectin concentration in the urine of patients with different levels of CFU was compared, it was found that the calprotectin concentration was significantly higher in the patients with high CFU (p = 0.007). In order to be able to make a more precise statement about the diagnostic ability of calprotectin, the subgroups were further divided. A total of 103 patients with pyuria were detected, of whom 72.82% (n = 71) had a CFU of ≥ 10^5^/ml. 38 patients with pyuria had a CFU of < 10^5^/ml. A comparison of the calprotectin concentrations in these two groups revealed a significantly higher calprotectin concentration in the group of patients with a CFU of ≥ 10^5^/ml.

12 patients had no pyuria but a CFU of ≥ 10^5^/ml and 47 patients a CFU of < 10^5^/ml. Patients with a CFU of ≥ 10^5^/ml had higher calprotectin concentrations, even in the absence of pyuria. The Mann–Whitney *U* test for the comparison of the calprotectin concentration in the urine of patients with UTI and those with a contamination with a CFU of ≥ 10^5^/ml showed a p-value of 0.492. There was therefore no significant difference in the calprotectin concentration in the urine between these two groups.

### Evaluation of the diagnostic accuracy of calprotectin in urine and pyuria for the detection of UTIs

**Figure 1:**
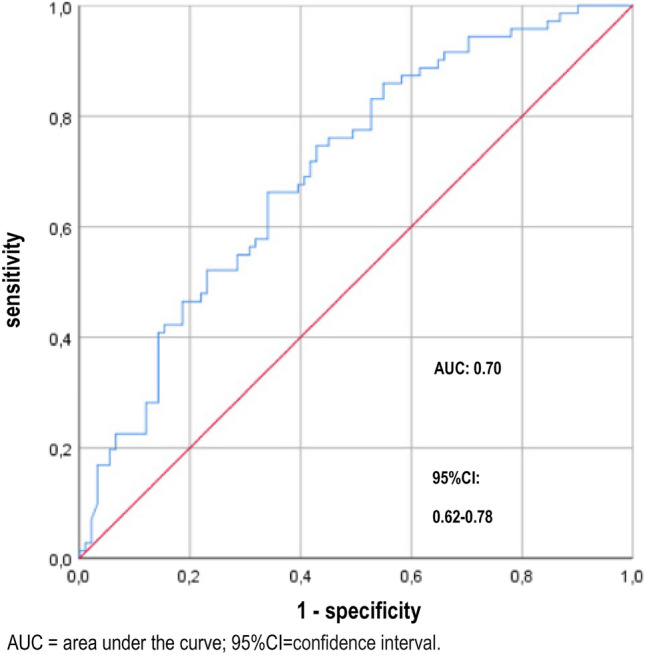
ROC curve of the calprotectin concentration in urine for the diagnosis of a UTI.

ROC curves were examined to analyse the diagnostic value of calprotectin as a marker for UTI. The ROC analysis of calprotectin in urine showed an AUC of 0.70 (Fig. 1). The threshold value for calprotectin in urine for the detection of a UTI was 1575 ng/ml. A sensitivity of 67.6% and a specificity of 59.3% were determined for this threshold value (95% confidence interval 0.62 to 0.78). The positive predictive value (PPV) was 56.5% and the negative predictive value (NPV) was 70.1%.

**Figure 2:**
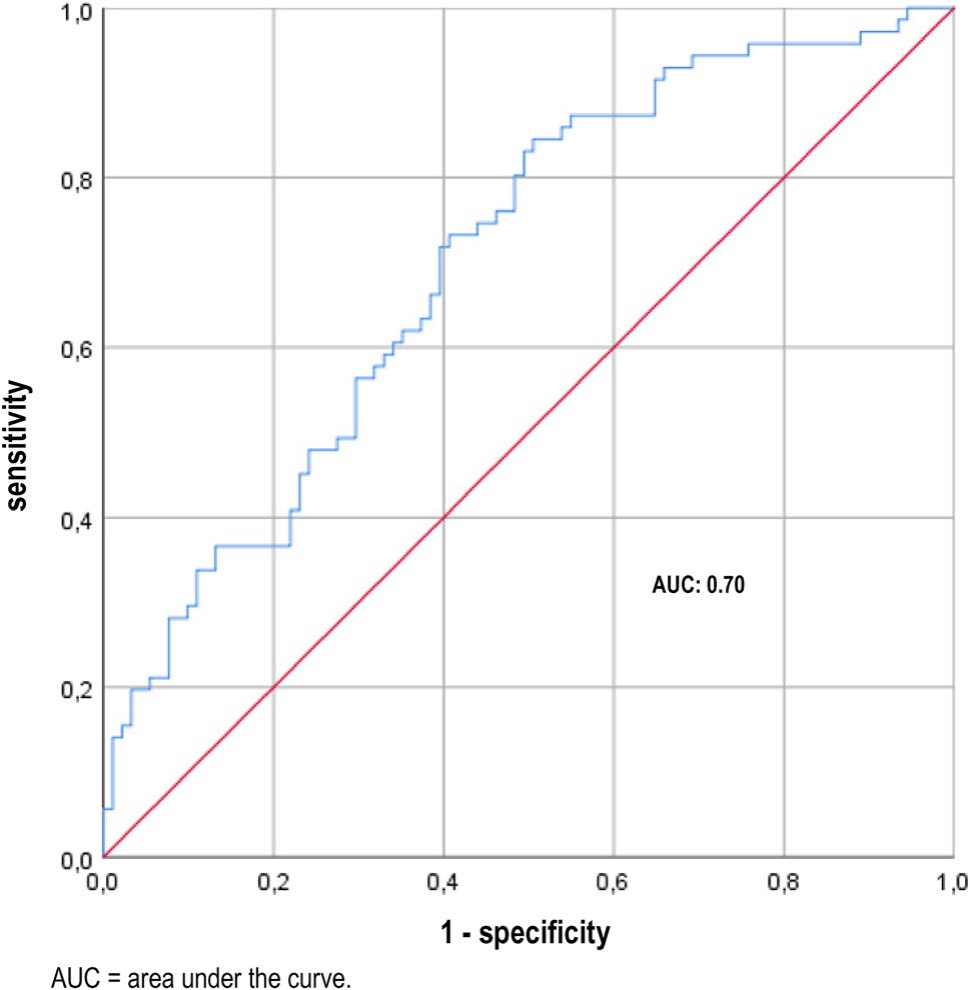
ROC curve of the calprotectin-creatinine quotient for the diagnosis of a UTI.

Figure 2 shows a ROC curve for the accuracy of the calprotectin-creatinine quotient for the detection of a UTI. In this ROC analysis, an AUC of 0.70 could be detected, i.e. the same quality for the detection of a UTI as by calprotectin in urine. The optimum threshold value was 2246 ng/mg creatinine. This cut-off value was associated with a sensitivity of 71.8% and a specificity of 60.4% (Fig. 2). The PPV was 58.6% and the NPV was 73.3%.

**Figure 3:**
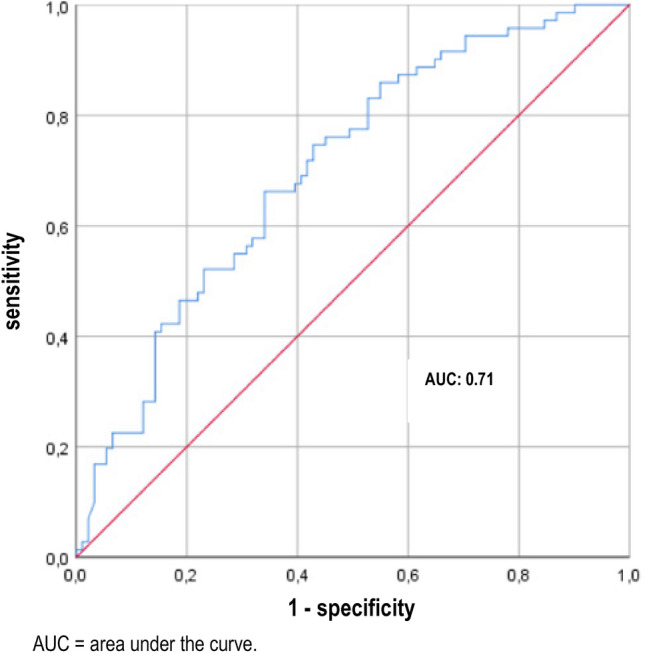
ROC curve of pyuria for the diagnosis of a UTI.

In order to further categorise the diagnostic value of calprotectin, an ROC analysis of pyuria was performed to detect a UTI. An AUC of 0.71 was calculated (Fig. 3). At a cut-off value of 12.5 leucocytes/µl, a UTI was detected with a sensitivity of 81.7% and a specificity of 51.6%. The PPV was 56.9% and the NPV 78.3%. The results are summarised in Table 5. By comparing the AUC of calprotectin and pyuria using the DeLong test it was shown that urinary calprotectin is not inferior to pyuria in detecting UTI (p = 0.9).

**Table 5:** Comparison of calprotectin concentrations in urine in different subgroups.

Subgroups	Calprotectin concentration ng/ml	*p*-value*
Pyuria 25/µl (median, IQR)	1684.2 (641.0–2681.9)	< 0.001
Pyuria 500/µl (median, IQR)	7074.8 (2192.5–19,114.9)	
CFU/ml 10^6^ (median, IQR)	3318.5 (1962.1–6000.2)	–
CFU/ml 10^4^ (median, IQR)	1862.1 (600.9–4550.5)	–
CFU/ml 10^5^ (median, IQR)	4614.4 (1183.8–8634.9)	0.007
CFU/ml 10^3^ (median, IQR)	787.8 (110.6–2061.4)	
CFU/ml < 10^5^ without pyuria (median, IQR)	95.6 (32.6–476.4)	0.029
CFU/ml ≥ 10^5^ without pyuria (median, IQR)	544.7 (74.9–2152.7)	
CFU/ml < 10^5^ with pyuria (median, IQR)	1900.2 (787.8–5661.5)	0.009
CFU/ml ≥ 10^5^ with pyuria (median, IQR)	5510.4 (242.4–6260.4)	
CFU/ml ≥ 10^5^ with contamination (median, IQR)	5010.3 (4800.4–15,996.8)	0.492
UTI (median, IQR)	3811.9 (1949.5–6847.9)	

*UTI* urinary tract infection, *CFU* colony forming units, *IQR* interquartile range;

*p-value between concentrations of calprotectin in urine in the subgroups from Mann–Whitney-*U*-Test.

## Discussion

The present study shows that urinary calprotectin is non-inferior to dipstick pyuria for the diagnosis of UTI. The desired superiority, however, could not be demonstrated. The typical markers of urine status were abnormal. The patients with a UTI had significantly more pyuria than those without a UTI (p < 0.05). Moreover, nitrite and proteinuria were more frequent in patients with UTI. All these findings are in line with prior findings^2,23–25^. *E. coli* was the most frequently detected uropathogen, followed by *Staphylococcus*, *Klebsiella pneumoniae*, *Enterococcus faecalis* and *Proteus mirabilis*. This spectrum is in line with former studies like ARESC (Antimicrobial Resistance Epidemiological Survey on Cystitis)^26^.

Considering the physiological origin of calprotectin—it is mainly found in leucocytes—an association of the urinary calprotectin concentration and the extent of pyuria appeared probable. Our findings support this hypothesis. This result is analogous to the results of the investigations of faecal calprotectin in the diagnosis of chronic inflammatory bowel diseases (IBD). The more severe the degree of infection, the higher the migration of leukocytes through the intestinal wall and the higher the concentration of faecal calprotectin^27^. As an established biomarker in gastroenterology, calprotectin is used to assess the diagnosis, disease activity and therapeutic outcomes of chronic IBD. Although calprotectin can differentiate between functional bowel symptoms and chronic IBD, it is not conclusive for differentiating among gastrointestinal infections and colorectal carcinomas^27^.

The ROC analysis had an AUC of 0.70. This value shows that there is indeed an association of urinary calprotectin concentrations but the diagnostic accuracy is limited. In analogy to pyuria, however, calprotectin was not able to differentiate symptomatic and asymptomatic bacteriuria. The AUC of calprotectin was almost identical to the AUC of pyuria (0.71). Using Youden Test based cut-off values the sensitivity and specificity values were comparable between the two approaches (67.60% and 59.30% vs. 81.7% and 51.6). In order to consider the grade of concentration of the urine, urinary calprotectin/creatinine ratios were calculated. The use of this ratio resulted in an almost unchanged AUC and only slightly improved sensitivy and specificity (59,3% and 60,4%). Thus, calculating this ratio appears dispensable.

In a review by Simmerville et al. from 2005, eight studies were analysed for the evaluation of pyuria for the detection of a UTI. As a result, a sensitivity of 72–97% and a specificity of 41–86% were determined for the detection of a UTI using pyuria in urine dipstick^8^. The results obtained here are therefore comparable with those described in the literature. A higher sensitivity and specificity for the detection of a UTI using urine dipstick is obtained by analysing the combination of pyuria and nitrite^28^.

In those 12 patients with UTI without pyuria, urinary calprotectin concentrations were indeed significantly higher than in those without UTI and without pyuria (p = 0.029). The present study was not powered, however, to compare the diagnostic potency of the two approaches in this subset of patients. This finding encourages a further study on this specific group of patients.

A biomarker should have causality to the disease in order to have diagnostic or prognostic value. To this point, the relationship between the biomarker and the disease must be consistent, coherent and specific. The occurrence of the biomarker should not be explained by other diseases or influenced by variables^29^. Another requirement is a precise and reliable measurement that can be performed repeatedly at low cost^30^. Calprotectin in urine as a biomarker for UTI does not sufficiently fulfil these criteria. It has previously shown that concentrations increase in AKI^16,17^ and urothelial malignancies^31^. Thus, calprotectin is no specific biomarker for UTI.

Several other urine biomarkers have been investigated for the detection of a UTI including myeloperoxidase (MPO), xanthine oxidase (XO), lactoferrin, urinary heparin-binding protein (UHBP), soluble triggering receptor expressed on myeloid cells-1 (TREM 1), and interleukins^32^. The present study adds a new biomarker on this list. In line with the other biomarkers, however, calprotecin is not superior to the cheap and broadly available dipstick examinations.

The study is limited by its sample size. Moreover, the diagnosis of UTI was based solely on the microbiological findings and not on the clinical findings. Formally, this is therefore an investigation of the diagnostic performance of biomarkers for the diagnosis of “significant bacteriuria”. However, this was also the intention, as urine culture as the gold standard can also only detect this entity. In other studies that analysed biomarkers in urine, a similar definition was used as in the present study.

## Conclusion

In conclusion, urinary calprotectin concentration is indeed a biomarker for UTI that is non-inferior to dipstick pyuria in ROC analyses. The lack of superiority to pyuria, however, does not justify its use in clinical practice. With regard to the findings in dipstick negative patients, it remains a candidate biomarker for symptomatic patients without pyuria. Future studies should focus on this specific group of patients.

### Supplementary Information

Supplementary Information.

## Data Availability

The datasets used in the current study are available from the corresponding author on reasonable request.
